# Community public health safety emergency management and nursing insurance service optimization for digital healthy urban environment construction

**DOI:** 10.3389/fpubh.2022.1032758

**Published:** 2022-10-18

**Authors:** Guosheng Hu, Zeyu Wang, Shaoxiang Jiang, Yuan Tian, Yue Deng, Yang Liu

**Affiliations:** ^1^School of Economics, South-Central Minzu University, Wuhan, China; ^2^School of Economics and Business Administration, Chongqing University, Chongqing, China; ^3^School of Public Administration, Guangzhou University, Guangzhou, China; ^4^National School of Development, Peking University, Beijing, China; ^5^Center for Enterprise Growth and National Economic Security Research, Tsinghua University, Beijing, China; ^6^Institute of Quality Development Strategy, Wuhan University, Wuhan, China; ^7^Population and Health Research Center, Zhongnan University of Economics and Law, Wuhan, China; ^8^Zhongnan Hospital of Wuhan University, Wuhan, China; ^9^School of Economics and Management, Wuhan University, Wuhan, China

**Keywords:** digital cities, health city, emergency management, nursing insurance system, community structure

## Abstract

The purpose of this paper is to promote the construction of digital healthy cities and improve the living standards of urban residents. Based on this, this paper analyzes the development of healthy cities, and studies community public health safety emergency management and nursing insurance service optimization methods for healthy urban environment construction. First, the concept of digital healthy urban environment construction is discussed. Then, the role of environmental health is discussed. Finally, two methods are designed to study the emergency management of public health safety and nursing insurance services in urban communities under the condition of environmental health. The results show that in the environmental health score of the city, the scores of X1 (the urban air quality excellent rate) and X6 (citizens' satisfaction with the environmental quality) were relatively low between 2016 and 2018, below 0.5 points. The scores for the remaining 3 years were relatively high, above 0.5. The scores of X2 (green coverage rate of built-up area), X3 (average grade sound effect of environmental noise in urban area), X4 (harmless treatment rate of domestic waste) and X5 (centralized treatment rate of domestic sewage) were relatively high from 2016 to 2018, above 0.5 points, and relatively low in the remaining 3 years, below 0.5 points. Meanwhile, residents are very satisfied with information collection and information management in public health and safety emergency management, and the number of very satisfied people is basically more than 40%. Satisfaction with resource allocation and privacy management is high, and the number of very satisfied people is basically above 30%. However, the satisfaction with risk perception and management measures is very low, and the number of very satisfied people is basically below 20%. It shows that the current construction of the community's public health and safety emergency management system is relatively poor in terms of X2–X5, while the development of X1 and X6 is relatively mature. The research not only provides a reference for the construction and improvement of a digital healthy city, but also contributes to the improvement of the healthy life of urban residents.

## Introduction

With the development of science and technology, digital and healthy cities have become important goals for future urban construction. The improvement of residents' life is crucial based on healthy city construction, and the healthy life of residents is the main purpose of healthy city construction (1). Based on this, the construction of a healthy city is mainly based on the living conditions of urban residents. Environmental health is the most critical issue affecting residents' living conditions, so improving urban environmental health plays a vital role in promoting the construction of healthy cities (2). In urban environmental health research, community public health safety emergency management and nursing insurance services are the most direct factors affecting residents' living standards (3). Although the current management of these factors is not perfect, many studies have provided references for its future development.

The development of digital healthy cities plays an important role in the overall development of society. The development of digital healthy cities can not only change the way of life of human beings and provide more convenience for human beings, but also promote the efficiency of urban construction and comprehensively promote social development. However, the current construction of public health safety emergency management and nursing insurance services in digital healthy cities is not perfect, so more research is needed to provide support for its development.

Based on this, this paper first discusses the construction needs and specific construction concepts of digital health cities. Then, the important role of urban environmental health in the construction of healthy cities is discussed. Finally, the public health security emergency management and nursing insurance service in a community are studied. The innovation is that it not only studies the development of digital healthy city, but also studies the comprehensive development of digital healthy city and community public health security emergency management system through the entropy weight method. The research not only provides a reference for community public health security emergency management and the optimization of nursing insurance services, but also contributes to the construction and development of healthy cities.

## Literature review

Although the current social achievements in the construction of the community public health security emergency management system in digital healthy cities are not outstanding, many studies have provided references for its development. First in foreign research, Sánchez (4) designed the architecture of the geospatial information sharing platform and the SOA (service-oriented architecture)-based spatial data sharing model based on the SOA architecture. Based on the development framework, the prototype system of the urban multi-source spatial information sharing platform is realized, which fully explains the development and design concept of the digital city (4). Park et al. (5), from the perspective of urban and regional informatization and digital city engineering construction, combined with digital city engineering practice, integrated urban construction field, 3S field, surveying and mapping science field, computer science field experts and scholars ' research results on digital city. A preliminary study on the basic concept of digital city, the theoretical framework of digital city, the engineering framework of digital city and other theoretical issues is made. The basic theoretical framework of digital city is put forward (5). Azzaoui et al. (6) pointed out that building healthy cities is a global action strategy advocated by the World Health Organization in the 1980s in response to the challenges to human health posed by urbanization. It aims to build a city that is constantly developing and developing its natural and social environment and expanding its social resources so that people can support each other in enjoying life and realizing their full potential (6). Rivani and Mei (7) pointed out that the good health of the population is the basic premise and condition of social and economic development, and public health is responsible for promoting and protecting the health of the population. Therefore, public health always adapts its strategies to promote and protect the health of the population according to the health problems faced by the population (7). To sum up, foreign research has entered a relatively mature stage for the construction of public health and safety emergency management system, and has formed relatively comprehensive research results. In addition, many studies in China have provided research references for public health security emergency management in digital health cities. Chen et al. (8) pointed out that under the influence of various factors, emergencies occur frequently, and public health emergencies are at the forefront, bringing many negative impacts on human life safety, social harmony and stability, and healthy economic development. Therefore, effectively responding to emergencies in the field of public health and strengthening research on public health emergency management have become one of the practical problems that governments at all levels need to solve urgently (8). Kang et al. (9) constructed an emergency management system framework for urban public health security based on data warehouse, emergency response support platform, unified management of information, and interconnection with other e-government systems, and elaborated on the basic functions of each business module and key technical methods for system implementation (9). Song et al. (10) pointed out that as the global aging process continues to accelerate, countries around the world have begun to establish long-term care insurance systems to deal with the long-term care risks of the elderly brought about by aging. Different countries have their own advantages and disadvantages in the system design of long-term care insurance due to differences in their own political environment, welfare system traditions, and cultural customs, especially in the design of long-term care insurance payment systems (10). Huang et al. (11) pointed out that the large scale of the elderly population and the continuously increasing and deeply aging population have led to a rapid increase in the number of disabled elderly people. Establishing a long-term care insurance system in line with the current social needs as soon as possible will undoubtedly become an important means to resolve the risk of disabled elderly care (11).

In summary, the current research on digital cities, public health security, emergency management and mutual insurance services in China and other countries has been very comprehensive. However, the practical application research of community public health emergency safety management and nursing insurance service in the construction of digital healthy cities has not yet appeared, so this paper is breakthrough research.

## Research theory and methods

### Construction of the digital healthy urban environment

#### Digital city

With the development of science and technology, digital construction has become the main direction of current social development. Digital city refers to the tendency of urban construction to be digitized. It is embodied in the informatization of the surveying, mapping, and statistical process of the earth's surface, the informatization of government management and decision-making process, the informatization of the people's life process, and the informatization of the management and decision-making process of enterprises in society (12). If urban construction can meet these four constructions needs, it will enter the era of urban informatization. Urban informatization construction is mainly based on computer, multimedia, and large-scale storage technology, with the broadband network as the link. Remote sensing, global positioning system, geographic information system, engineering measurement technology, simulation-virtual technology, and other technologies are used to carry out multi-resolution, multi-scale, multi-space-time, and multi-type three-dimensional descriptions of cities. Information technology means are used for digitizing and virtualizing all the content of the city's past, present, and future on the network (13).

Specifically, the digital city refers to the use of spatial information to build a virtual platform and to obtain and load urban information in digital form, including urban natural resources, social resources, infrastructure, humanities, and economy, to provide a wide range of services for the government and all aspects of society. Digital cities can realize comprehensive analysis and effective utilization of city information. It supports urban planning, construction, operation, management, and emergency response through advanced information technology. It can effectively improve government management and service levels, enhance urban management efficiency, save resources, and promote sustainable urban development. On the one hand, 80% of the content in human life and the production process is related to urban space and its location, so the digital city is constructed and operated based on the spatial information platform (14). On the other hand, the spatial information platform is the infrastructure construction in the process of digital city construction. Various high-end digital city applications need to be realized through the spatial information platform and are restricted by the construction of the spatial information platform. Therefore, the relationship between spatial information platforms and digital cities is very close. In building a digital city, it is necessary to consider constructing a spatial information platform (15).

The speed of urban development is getting fast based on the construction of information cities. The quality of urban construction is also constantly improving, so the role of information city construction is vital.

#### Healthy city

With the continuous acceleration of urbanization, the demand for urban construction is also increasing. In the current urban construction, a healthy city is an important indicator to regulate the quality of urban construction (16). According to statistics, about 60% of people will live in big cities by the middle of the 21st century. The increasing number of people in cities will bring many problems to urban construction, including environmental pollution, traffic jams, high unemployment, and housing shortages (17). These problems can affect the progress of urban construction, the final quality, and the health of residents. Therefore, in the current urban construction, a healthy city has also become one of the main goals of urban construction (18).

The healthy city is the concept of urban construction proposed by the WHO at the end of the 20th century. A healthy city mainly refers to the three main factors of urban construction to meet residents' health, environmental health, and social health. In the construction of a healthy city, the realization of these three goals may be in line with the construction of a healthy city. The specific operation is to improve residents' health, improve the urban environment's condition, and optimize the utilization of social resources (19). Healthy residents are urban residents who are in a healthy state. A healthy urban environment refers to the environment in the city that can ensure the healthy life of urban residents. It guarantees the physical and psychological healthy living needs of urban residents. Healthy social resources refer to the rational use of social resources to ensure urban residents' healthy life and development (20).

Based on the above theories, a healthy digital city is a new type built by combining digital and healthy city concepts. A healthy digital city can satisfy the digital development of the city, improve the effect of urban development and the efficiency of management, and provide support for the healthy development of the city.

### Analysis of the role of environmental health

Environmental health is an important indicator for building healthy cities. Environmental health refers to ensuring the healthy growth of urban residents in urban construction. The urban environmental health evaluation method has five aspects to the standard proposed by the WHO. In the China Research Society of Urban Development (CRSUD), the evaluation of environmental health is different (21). Table 1 shows the urban environmental health evaluation system of the WHO and the CRSUD (22).

**Table 1:** The urban environmental health evaluation system of the WHO and the CRSUD.

**Organization**	**First-level indicator**	**Secondary indicators**
WHO	Industry (W1)	Air pollution (W11)
		Water quality (W12)
		Sewage treatment rate (W13)
	Life (W2)	Domestic garbage collection (W21)
		Domestic waste disposal (W22)
	Construction (W3)	Green space coverage (W31)
		Green space accessibility (W32)
		Vacant industrial land (W33)
	Infrastructure (W4)	Sports and leisure facilities (W41)
		Sidewalk (W42)
		Bicycle lane (W43)
	Public works (W5)	Public transit (W51)
		Coverage of the public transport network (W52)
		Living space (W53)
China Urban Development Research Association	Basic environment (W1)	Air quality compliance days (W11)
		Centralized treatment rate of urban sewage (W12)
		Harmless treatment rate of domestic waste (W13)
		Greening rate of built-up area (W14)
		Urban population density (W15)
	Cultural environment (W2)	The number of theaters and theaters per square kilometer in the urban area (W21)
		The total number of books in public libraries per thousand people (W23)
		Internet penetration (W23)
	Social conditions (W3)	Number of medical institutions per square kilometer in urban areas (W31)
		Hospital and health care beds per 1,000 people (W32)
		Number of practicing (assistant) physicians per thousand people (W33)
		Number of people insured by basic medical insurance (W34)

From Table 1, the WHO has proposed an evaluation system for environmental health in healthy cities, similar to the environmental health evaluation system proposed by the CRSUD. However, the evaluation system of the WHO mainly includes the basic environmental evaluation of the city. The evaluation system proposed by the CRSUD contains comprehensive contents (23). The ultimate goal of these evaluations is to provide references for building a healthy urban environment, thereby providing important support for the healthy life of urban residents.

### Urban community public health safety emergency management and nursing insurance service under environmental health

The urban community is the smallest unit of urban management and an important object in urban construction. The concept of urban residents' autonomy can be well realized based on the urban community (24). Public health security emergency management and mutual insurance services are serious problems facing urban communities. These problems will cause damage to the health of the urban environment and affect the health of residents. Public health security directly affects the health of residents, and it has the characteristics of great harm, poor controllability, poor persistence, and high complexity (25). As a result, the response to public health events should not be limited to the field of public health, and the causes of events and the particularity of their hazards should be comprehensively analyzed. Based on this, targeted emergency management work is conducted to minimize the impact of public health emergencies on the country, society, and individuals, ensure the safety of human life and property, and maintain social stability and harmonious development (26).

Emergency management refers to a series of management activities to ensure public safety, control the situation, and reduce losses when a public emergency occurs (27). Community emergency management capability is one of the essential modules of the government's public management system. Improving community emergency management capacity is critical to improving government administrative capacity. It is also an indispensable and important guarantee to enhance the sense of urban security and improve people's happiness (28).

Nursing care insurance refers to insurance that provides compensation for the cost of nursing services for those insureds who need long-term care due to old age, illness, or disability (29). With the continuous improvement of urbanization, the demand for nursing care insurance services is increasing rapidly. The needs for nursing services mainly include the nursing needs of the elderly, post-critical illnesses, and the disabled. Therefore, improving the quality of community nursing services to improve urban environmental health cannot be ignored (30).

Based on the above discussion, community public health safety emergency management and nursing insurance service are two very important parts of the optimization of urban environmental health. Therefore, this paper designs and evaluates the current situation of community public health safety emergency management and nursing insurance service in the current urban environmental health optimization to provide a reference for future community public health safety emergency management and nursing insurance service optimization.

### Community public health safety emergency management and nursing insurance service evaluation method

Based on the above theories, a comprehensive investigation and analysis of a community's urban environmental health transition are carried out. Research and analysis of the changes in community public health safety emergency management and nursing insurance services are also conducted. The specific methods used are the entropy weight method and questionnaire survey method. The entropy weight method research needs to determine the research object set as *X*, and its evaluation matrix is:


(1) $$\begin{array}{l} X={\left(X_{ij}\right)}_{n\times m} \end{array}$$

In Equation (1), *n* represents the number of evaluation objects, and *m* represents the number of evaluation indicators. *i* and *j* mean the position of the evaluation object in the matrix (31). The data is standardized, and the positive indicator is processed according to Equation (2).


(2) $$\begin{array}{l} Z_{ij}=\frac{X_{ij}-X_{min}^{i}}{X_{max}^{i}-X_{min}^{i}} \end{array}$$

In Equation (2), *Z*_*ij*_ represents the standard value, $X_{\min}^{i}$ represents the minimum value of the original data, and $X_{\max}^{i}$ represents the maximum value of the original data. Inverse indicator data can be processed according to Equation (3).


(3) $$\begin{array}{l} Z_{ij}=\frac{X_{max}^{i}-X_{ij}}{X_{max}^{i}-X_{min}^{i}} \end{array}$$

Then, the weight of the data is calculated.


(4) $$\begin{array}{l} P_{ij}=Z_{ij}/\sum_{i=1}^{m}Z_{ij} \end{array}$$

All elements in the Equation (4) have the same meaning as the above equations. Then the entropy value of the data is calculated, and the calculation equation is:


(5) $$\begin{array}{l} e_{j}=-k\sum_{i=1}^{m}\left(P_{ij}\;*\;\ln\;P_{ij}\right) \end{array}$$

In Equation (5), *k*>0. Besides, the coefficient of variance is calculated for the data.


(6) $$\begin{array}{l} d_{j}=1-e_{j} \end{array}$$

Finally, the weights of the data are calculated.


(7) $$\begin{array}{l} w_{j}=d_{j}/\sum_{j=1}^{n}d_{j} \end{array}$$

**Figure 1:**
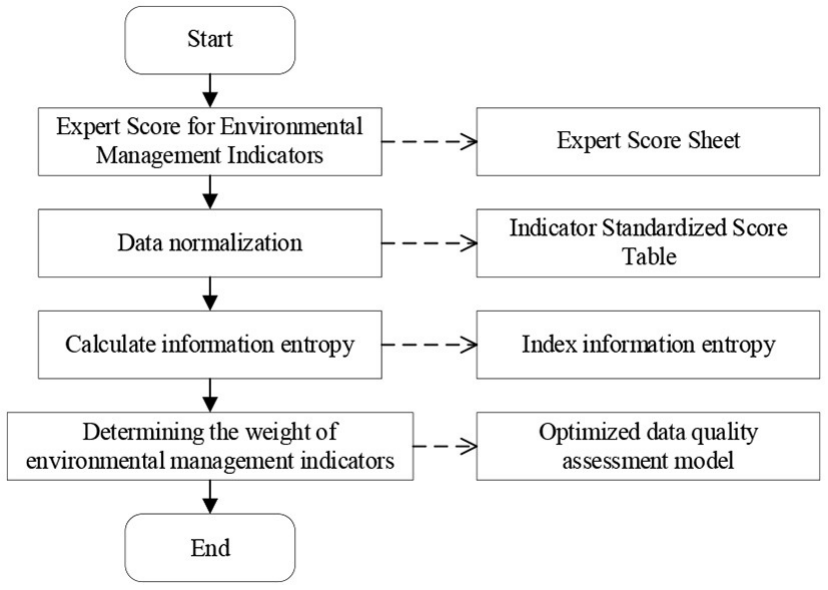
Community public health safety emergency management evaluation process.

Based on the above analysis, an investigation and evaluation of the urban environmental health status of a community is conducted. Then, through a questionnaire survey, the community public health safety emergency management and nursing insurance service status are investigated and evaluated (32, 33). Figure 1 shows the specific process of community public health safety management evaluation using the entropy weight method.

Figure 1 shows that this paper mainly evaluates the community public health safety emergency management system through the entropy weight method. Table 2 shows the specific content of the questionnaire survey method.

**Table 2:** Questionnaire survey information.

**Classification by**	**Classification**	**Number of people**	**Proportion (%)**
Gender	Male	200	50
	Female	200	50
Age	18–30	92	23
	30–40	112	28
	40–50	124	31
	50–60	72	18

Table 2 shows that the surveys and evaluations of community users are based on gender and age. Age classification starts from 18 years old and conforms to the basic norms of the questionnaire. A total of 400 questionnaires are distributed, and 392 valid questionnaires are recovered, with an effective rate of 98%, which is in line with the norms of the questionnaire survey. Therefore, the research is valid. The subject of the questionnaire is to evaluate the public health security emergency management and nursing insurance services in the community.

**Figure 2:**
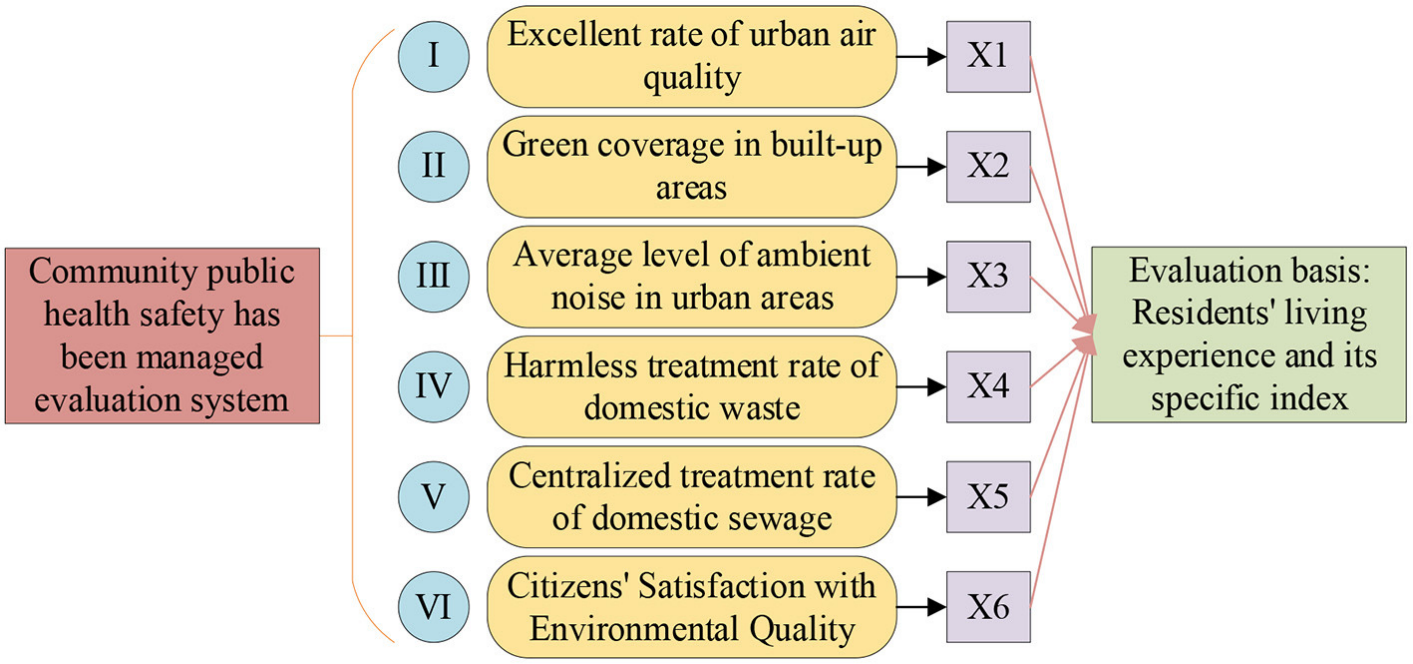
Basic information on urban environmental health assessment.

The design evaluates the urban construction in which a community is located, mainly to investigate and evaluate the environmental health status of the digital healthy city. Among them, the environmental health assessment includes the assessment of urban infrastructure and services. The evaluation system is made according to the evaluation indicators of WHO and China Urban Development Research Association in Table 1, and constitutes six indicators X1–X6. They are the excellent rate of urban air quality, the green coverage rate in built-up areas, the average level of ambient noise in urban areas, the harmless treatment rate of domestic waste, the centralized treatment rate of domestic sewage, and the citizens' satisfaction with environmental quality. Figure 2 shows the basic information of a city's environmental health assessment.

Figure 2 shows that the research contents include the excellent rate of urban air quality, the green coverage rate in built-up areas, the average level of ambient noise in urban areas, the harmless treatment rate of domestic waste, the centralized treatment rate of domestic sewage, and the citizens' satisfaction with environmental quality. The main evaluation basis includes residents' feelings and specific parameters of various indicators. Based on the above basis, this paper conducts a comprehensive survey on the environmental health status of the city where a community is located. First, the changes of various indicators of the city are counted, and the statistical method is the coefficient of variation of the city's recognition of the indicator when it was formulated to determine the specific basis for the city's environmental health assessment.

## Assessment of results

### Digital health city environmental health assessment

**Figure 3:**
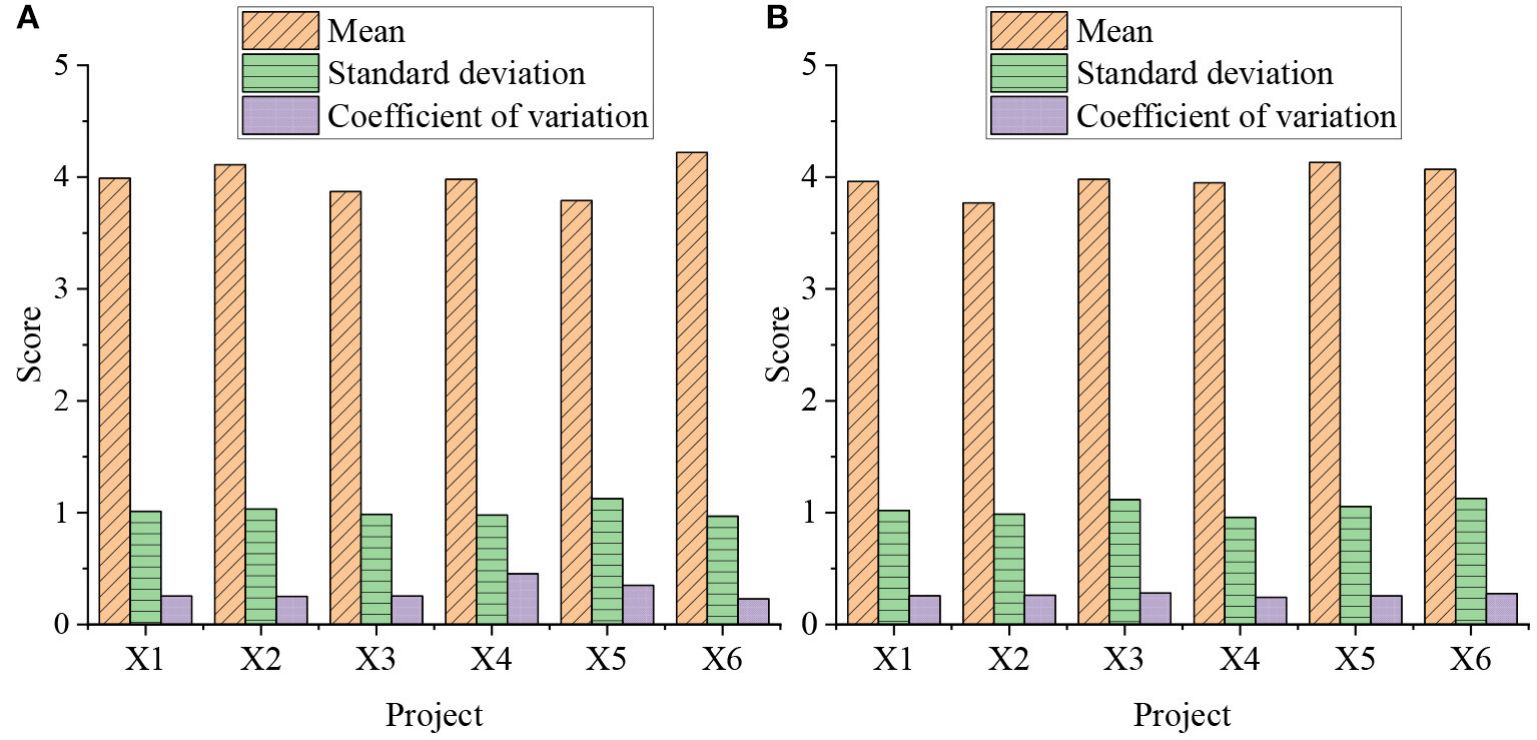
The specific parameters of the city's evaluation of each indicator in 2020 and 2021 [**(A)** is 2020; **(B)** is 2021].

Through the evaluation system established above, the current situation of community public health safety emergency management and nursing insurance services is evaluated. Figure 3 shows the specific parameters of the city's evaluation of each indicator in 2020 and 2021.

**Figure 4:**
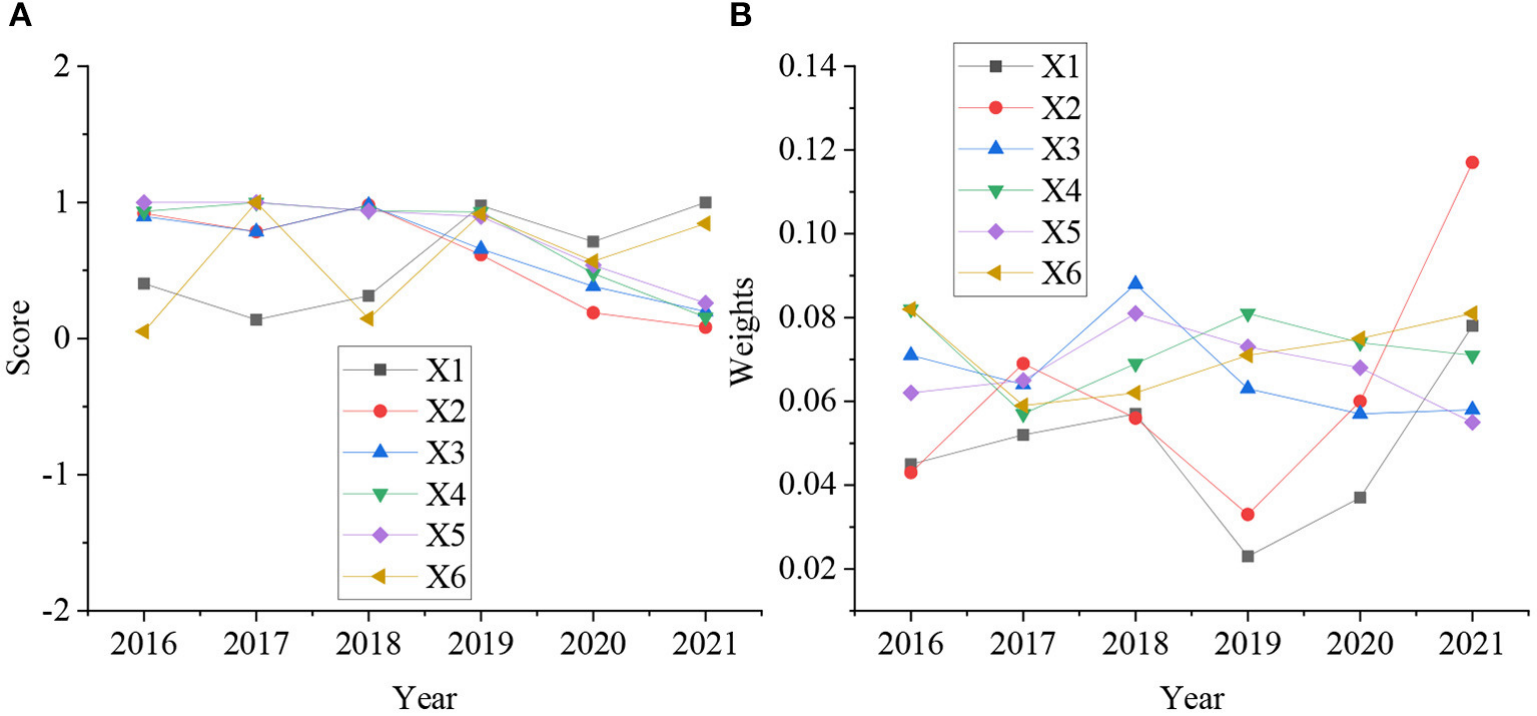
Evaluation results of urban environmental health scores and weights [**(A)** is environmental health assessment; **(B)** is environmental health weight].

Figure 3 shows that in the establishment of the urban environmental health assessment index, the average scores of the indicators such as the excellent and good rate of air quality in the urban area of the city in 2020 and 2021, the green coverage rate in built-up areas, the average level of ambient noise in urban areas, the harmless treatment rate of domestic waste, the centralized treatment rate of domestic sewage, and the citizens' satisfaction with environmental quality are all around 4 points. It shows that it is reasonable to evaluate the environmental health construction of the city through the excellent rate of urban air quality, the green coverage rate in built-up areas, the average level of ambient noise in urban areas, the harmless treatment rate of domestic waste, the centralized treatment rate of domestic sewage, and the citizens' satisfaction with environmental quality. Figure 4 shows the scoring and weight evaluation results of various indicators of the city.

From Figure 4, through the evaluation, it is found that the scores of X1 and X6 in the environmental health score of the city are relatively low between 2016 and 2018, below 0.5. The scores for the remaining 3 years are relatively high, above 0.5. The scores of X2, X3, X4, and X5 are relatively high from 2016 to 2018, above 0.5. The remaining 3 years are relatively low, below 0.5. The weights of all indicators are above 0.4 every year.

### Community public health safety emergency management and nursing insurance service optimization evaluation

**Figure 5:**
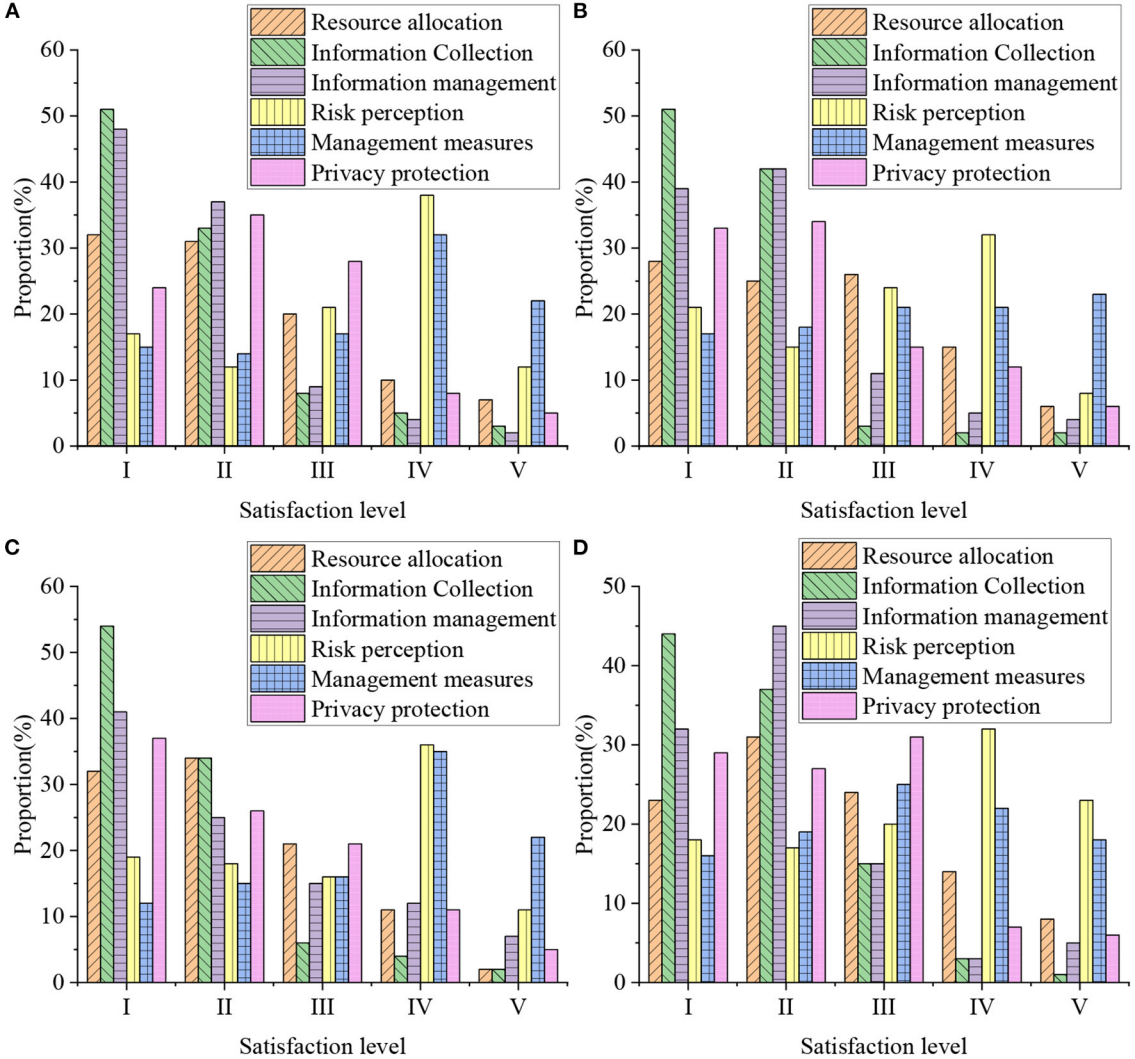
Evaluation results of community health and safety emergency management [**(A)** is 18–30 years old, **(B)** is 30–40 years old, **(C)** is 40–50 years old, and **(D)** is 50–60 years old].

In terms of emergency management of social public health security, this paper mainly evaluates the management of the COVID-19. The main assessment is based on the emergency management situation during the disaster, including factors such as resource allocation, information collection, information management, risk perception, management measures, and privacy protection during the epidemic. Therefore, this paper conducts research and evaluation on the residents of a certain community based on this evidence through a questionnaire survey. The evaluation of the community's public health security emergency management status is mainly based on the residents' satisfaction, and then provides a reference for the optimization of community public health security emergency management. In the research, the user classification in Table 2 is mainly used as the standard to investigate and the current public satisfaction is evaluated. Figure 5 shows the results of the assessment of community health security emergency management.

In Figure 5, I–V represent very satisfied, satisfied, general, dissatisfied and very dissatisfied, respectively. In the evaluation of community public health security emergency management, residents are very satisfied with the information collection and information management in public health security emergency management, and the number of very satisfied people is basically more than 40%. Satisfaction with resource allocation and privacy management is high, and the number of very satisfied people is basically above 30%. However, the satisfaction with risk perception and management measures is very low, and the number of people who are very satisfied is basically below 20%. It indicates that at present, the optimization of public health safety management in this community needs to focus on risk perception and management measures. Meanwhile, it is necessary to strengthen the improvement and maintenance of other aspects.

### Digital health city nursing insurance service optimization measures

**Figure 6:**
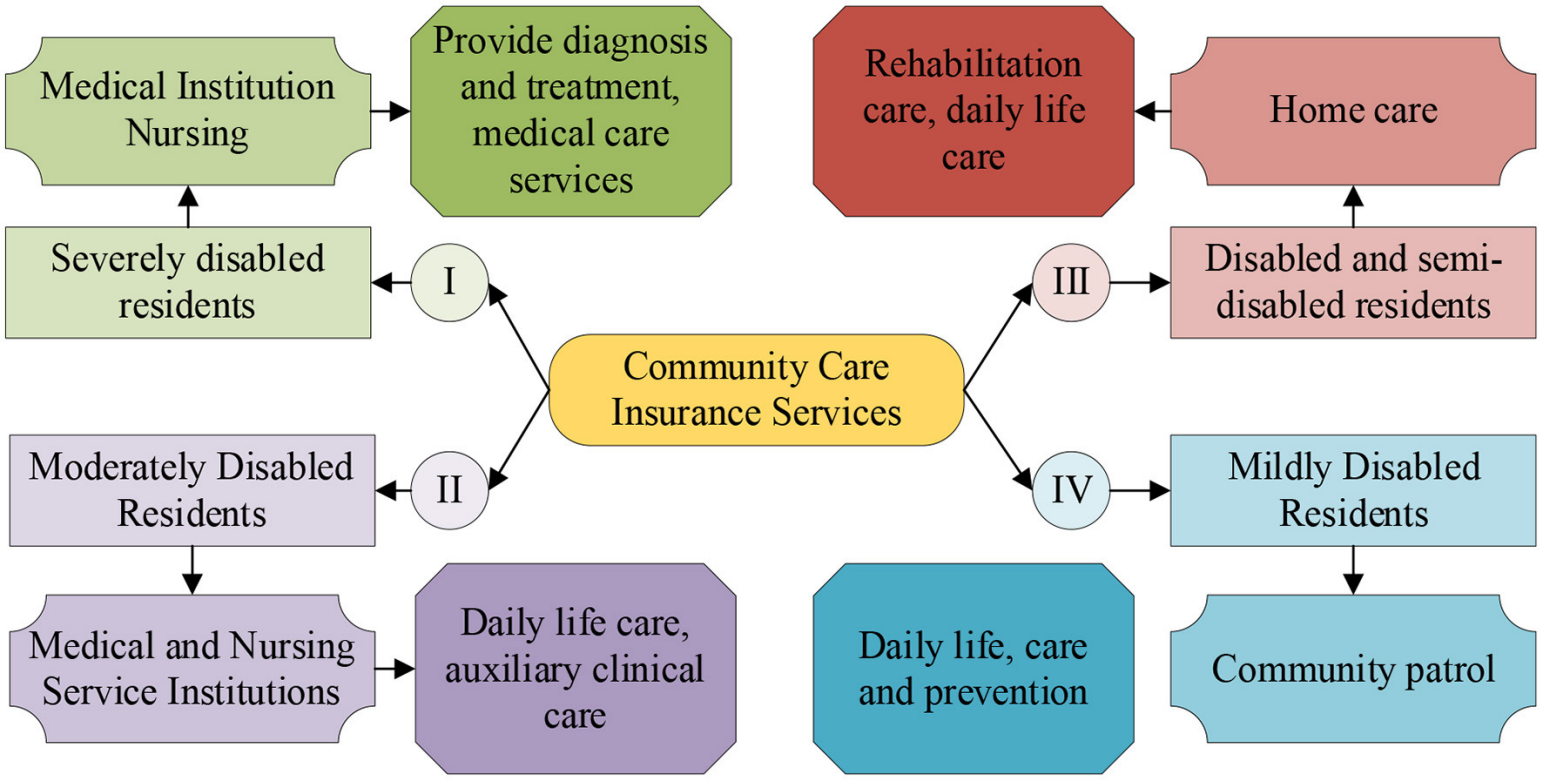
Optimal design of community nursing insurance services.

Based on the optimization of community nursing insurance services, Figure 6 shows the optimized design of community nursing insurance services.

Figure 6 shows that, based on the survey of residents in this community, the current community nursing insurance service has not been fully established. Therefore, in order to optimize the nursing insurance service in this community, a more comprehensive community nursing insurance service design is proposed for the community. First, the groups of residents who need nursing insurance services are classified into four levels in total, namely severely disabled residents, moderately disabled residents, disabled and semi-disabled residents, and mildly disabled residents. And different community nursing insurance service projects and methods are designed for residents of different levels to provide a reference for the overall optimization of nursing insurance services in the community. Meanwhile, it also provides support for the overall community care insurance services in the city. As mentioned above, this paper comprehensively studies the current situation of public health emergency safety management and nursing insurance services in digital healthy urban communities, and conducts research on their optimization, thus providing an important reference for the construction and development of digital healthy cities across the country.

## Conclusion

With the continuous development of urban construction, a digital healthy city has become an important goal of urban development. Therefore, in order to promote the development of healthy cities and improve the living conditions of urban residents, this paper first discusses the construction process of digital healthy cities. Secondly, the important role of environmental health in the improvement of residents' lives in digital health cities is discussed. Finally, through two methods, namely questionnaire survey and entropy weight method, research on environmental health and community public health security emergency management and community nursing insurance service in a city is designed. The research results show that the environmental health scores of X1 and X6 in the city were relatively low between 2016 and 2018, below 0.5, and the scores in the remaining 3 years were relatively high, above 0.5. The scores of X2, X3, X4, and X5 were relatively high from 2016 to 2018, above 0.5 points, and the remaining 3 years were relatively low, below 0.5 points. And the weight value of all indicators is above 0.4 every year. Residents are very satisfied with information collection and information management in public health and safety emergency management, and the number of very satisfied people is basically more than 40%. Satisfaction with resource allocation and privacy management is high, and the number of very satisfied people is basically above 30%. However, the satisfaction with risk perception and management measures is very low, and the number of very satisfied people is basically below 20%. Meanwhile, this paper also provides guidance on the emergency management of public health security in the urban community and the optimization of community nursing insurance services. Although a relatively comprehensive study of the environmental health status of a city, community public health emergency safety management and nursing insurance service status has been provided, and important guidance has been provided, the overall study of the city is not comprehensive enough. Therefore, in the future, the research scope will be expanded, and the construction of the public health security emergency safety management system in the process of digital city construction will be optimized to provide reference for the future construction of digital healthy cities.

## Data availability statement

The original contributions presented in the study are included in the article/supplementary material, further inquiries can be directed to the corresponding authors.

## Author contributions

GH and ZW: conceptualization, validation, and supervision. YD and GH: methodology, project administration, and funding acquisition. YL and SJ: software. YD and YL: formal analysis. SJ and GH: investigation. SJ and YT: resources. YT and GH: data curation. GH, ZW, and YD: writing—original draft preparation and writing—review and editing. YL: visualization. All authors contributed to the article and approved the submitted version.

## Funding

This work was supported by Youth Project of Humanities and Social Science of the Ministry of Education, China (Grant No. 18YJC790049) and the Philosophy and Social Science Research Project in Department of Education of Hubei Province (Grant No. 21G001).

## Conflict of interest

The authors declare that the research was conducted in the absence of any commercial or financial relationships that could be construed as a potential conflict of interest. The handling editor B-JH declared a shared affiliation with the authors GH at the time of review.

## Publisher's note

All claims expressed in this article are solely those of the authors and do not necessarily represent those of their affiliated organizations, or those of the publisher, the editors and the reviewers. Any product that may be evaluated in this article, or claim that may be made by its manufacturer, is not guaranteed or endorsed by the publisher.
